# Assessment of the effectiveness of West Nile virus screening by analysing suspected positive donations among blood donors, Germany, 2020 to 2023

**DOI:** 10.2807/1560-7917.ES.2025.30.8.2400373

**Published:** 2025-02-27

**Authors:** Stefano Orru', Annette Reissinger, Angela Filomena, Anna Heitmann, Markus Benedikt Funk, Jonas Schmidt-Chanasit, Julia Kreß, Heinrich Scheiblauer, Dániel Cadar, Sarah Anna Fiedler

**Affiliations:** 1 Division of Safety of Biomedicines and Diagnostics, Paul-Ehrlich-Institut, Langen, Germany; 2 Section of Molecular Virology, Paul-Ehrlich-Institut, Langen, Germany; 3 Testing Laboratory for In Vitro Diagnostics, Paul-Ehrlich-Institut, Langen, Germany; 4 Department of Arbovirology and Entomology, National Reference Center for Tropical Infectious Pathogens, Bernhard Nocht Institute for Tropical Medicine, Hamburg, Germany; 5 Faculty of Mathematics, Informatics and Natural Sciences, University of Hamburg, Hamburg, Germany

**Keywords:** West Nile virus, blood donor screening, next-generation sequencing, nucleic acid amplification technique, antibody testing, risk minimisation measure, safety of blood and blood components

## Abstract

**Background:**

The first autochthonous human West Nile virus (WNV)-positive cases in Germany were confirmed in 2019. Risk minimisation measures (RMM) were introduced in 2020; no WNV transfusion-transmitted infections have been reported to date.

**Aim:**

To analyse German suspected WNV-positive blood donations during annual seasons 2020–23 to review donor testing requirements.

**Methods:**

WNV look-back procedures were initiated as per German regulations and additional donor data were collected. Blood samples were analysed by metagenomic next-generation sequencing (mNGS), individual donor nucleic acid amplification technique (ID-NAT)-based testing and antibody (Ab) testing.

**Results:**

Seventy-four cases were followed up after WNV-positive donor mini-pool screening. Forty-five (83%) of 54 samples tested with the cobas WNV assay and 14 (29%) of 49 samples tested with the RealStar WNV assay showed a reactive ID-NAT-based result; the viral load ranged between 70,251 IU/mL and values below quantification limits. Fifteen (23%) of 64 samples serologically tested were reactive with at least one of the three Ab tests performed; the previous WNV-negative donation was nearly always documented > 28 days before. Of 73 samples sequenced, mNGS detected WNV in 26 (36%) and other flaviviruses in 14 (19%) cases.

**Conclusion:**

In some suspected cases where a WNV infection was not confirmed, mNGS demonstrated a cross-reaction with other flaviviruses. Ab testing could only detect WNV in late stages of infection. A NAT-based WNV donor screening with a detection limit of at least 120 IU/mL seems to be a sufficiently effective RMM at present. However, a continuous re-evaluation of test strategy is always required.

**Key public health message**

**What did you want to address in this study and why?**

In humans, West Nile virus (WNV) can be transmitted through blood transfusion from infected donors and be life-threatening in rare cases. Blood donor deferral and screening were implemented in Germany to reduce this risk. We analysed epidemiological data and tested blood samples of suspected WNV-positive donors from 2020 to 2023 to assess the effectiveness of current national safety measures.

**What have we learnt from this study?**

We found that WNV was endemic in several German federal states and that blood establishments (BE) could detect WNV-positive blood donations even at low virus concentrations. However, not all suspected cases were confirmed and, depending on the sensitivity of the specific diagnostic tool used for screening, it was not always possible for BE to distinguish between WNV and other viruses belonging to the same family.

**What are the implications of your findings for public health?**

Although there have never been WNV transfusion-transmitted infections so far in Germany, this risk should be continuously monitored. Accurate documentation of national endemic areas is helpful to target risk minimisation measures. Furthermore, the currently prescribed screening strategy adopted by BE appears to be sufficiently reliable to detect WNV-positive donors and thus ensure safe blood transfusions.

## Introduction

West Nile Virus (WNV) belongs to the *Flavivirus* genus and shares structural and genetic similarities with other medically important flaviviruses, such as Dengue virus (DENV), Zika virus (ZIKV) and Japanese Encephalitis virus (JEV) [1]. It is classified into at least nine genetic lineages, of which lineages 1 (L1) and 2 (L2) are the most relevant to human disease and are widely distributed [2]. In Europe, WNV L2 has become dominant in recent years, contributing to more frequent and larger outbreaks, as well as endemic transmission in several countries, particularly in southern and central Europe [3].

WNV is mainly transmitted to humans through mosquito bites. *Culex* mosquitoes represent the principal vector that transmits WNV from avian reservoir hosts to horses and humans [4]. Other transmission routes to humans are organ transplantation and blood component transfusion from WNV-positive asymptomatic donors. WNV vaccines are available for equids [5], but none are approved for human use [6]. One in five (20%) human WNV infections manifest a West Nile fever and/or influenza-like symptoms, while the remaining infections (80%) remain asymptomatic [7,8]. Ca 1% of infections develop into severe West Nile neuroinvasive disease, particularly in high-risk groups like older people aged over 70 years and immunocompromised individuals [9-11]; on average, 9% of West Nile neuroinvasive disease cases have a fatal outcome [12-14].

According to section seven of the German Infection Protection Act and section 22 of the German Transfusion Act, healthcare facilities, such as surgeries or hospitals, and operators of blood establishments (BE) are obliged since 1998 to report human cases of suspected WNV transmission through blood transfusion to the specific regional competent authority and to the Robert Koch Institute (RKI), as national competent authority for infectious diseases and non-communicable diseases. The screening of blood donations for WNV and other flaviviruses began in Germany even before the first confirmed autochthonous human WNV cases were registered in 2019 [15-17]. In June 2020 in response to the detection of the first cases, the national competent authority for vaccines and biomedicines, Paul-Ehrlich-Institut (PEI), which is responsible for the monitoring, analysis and prevention of the risks of a WNV infection associated with blood component transfusion in Germany, updated the national graduated plan procedure for handling WNV based on risk level. German BE currently select one among the following risk minimisation measures (RMM) [18]: (i) 4-week deferral for blood donors who stayed for 2 consecutive days within the WNV season (1 June–30 November) in a WNV endemic area listed on the PEI online database of emerging infections [19]; (ii) pathogen reduction of platelets and plasma through a certified market-approved technology [20,21]; (iii) certified market-approved or (iv) in-house nucleic acid amplification technique (NAT)-based testing of blood components that have for a single donation an analytical sensitivity expressed as WNV RNA limit of detection (LoD) of at least 120 IU/mL for both WNV L1 and L2 [22].

The aim of the study was to obtain an in-depth overview of the suspected WNV-reactive cases in Germany over the 2020–23 seasons. The focus was on frequency, epidemiology, time of onset and localisation. This allowed an up-to-date evaluation of the effectiveness of the German RMM adopted to prevent WNV transfusion-transmitted infections, especially concerning the minimum analytical sensitivity for NAT-based systems.

## Methods

### Pooled and individual nucleic acid amplification technique-based testing

Before releasing blood components for transfusion, WNV screening is performed independently at each BE to ensure its safety. The German WNV graduated plan procedure states that BE can test for WNV using at least one NAT-based system among a different selection of certified market-approved screening tests or non-screening tests requiring in-house validation.

In general, blood samples from different donors are pooled for screening for efficiency reasons. The maximal mini-pool (MP) size in which BE can pool blood samples from different donors depends on the LoD of the NAT-based method employed, considering the mandatory required value of at least 120 IU/mL for a single donation. Reactive MP-NAT results require a verification by a more sensitive individual donor (ID)-NAT. 

### Look-back procedure

In case of reasonable grounds to suspect WNV infections, BE initiate a look-back procedure which includes measures taken to trace and protect both donors and recipients as described in section 63i of the German Medicinal Products Act and section 19 of the German Transfusion Act. The corresponding donors and possible related blood components are deferred until the suspected WNV infection cases can be clarified [23].

As part of the look-back procedure, PEI receives a notification form from the BE that collected the NAT screening result, containing general information about the blood donor (e.g. sex, age, donation frequency, date of suspected WNV-positive donation and of previous WNV-negative donation, etc.) set out by section 14 of the German Transfusion Act. If applicable, PEI sends out a standardised questionnaire asking for further details regarding NAT screening (e.g. system used, analytical sensitivity, MP size, viral concentration detected, etc.) and blood donor (e.g. residence district, stay or not stay in WNV-affected area, etc.) for study purposes.

### Metagenomic next-generation sequencing

Samples of suspected WNV-positive blood donations are forwarded from BE to Bernhard Nocht Institute for Tropical Medicine (BNITM), which serves as the reference laboratory for WNV in Germany. Unbiased metagenomic next-generation sequencing (mNGS) is employed to conduct further investigations into samples NAT-tested WNV-reactive or false reactive by BE. The goal is to determine the WNV lineage and/or detect any potential infection with other flaviviruses.

The RNA extracted and purified from plasma or serum samples undergoes deep sequencing using an in-house mNGS pipeline established for virus discovery. Following processing, the normalised libraries are sequenced using a 200-cycle (2x100 bp paired-end) reagent kit (Illumina Inc.) on a NextSeq 2000 platform (Illumina Inc.). The employed mNGS pipeline is capable of detecting DNA or RNA viruses at a minimum concentration of 10 copies/µL.

### Individual donor nucleic acid amplification technique-based testing

To corroborate their own NAT results, BE were asked to send additional aliquots of suspected WNV-reactive donor samples to PEI as the responsible authority for blood safety in Germany, which performed further ID-NAT tests with two different platforms, the cobas WNV for use on the cobas 6800/8800 systems (Roche Diagnostics Inc.) and the RealStar WNV RT-PCR Kit 2.0 (altona Diagnostics GmbH).

The cobas WNV is a qualitative in vitro nucleic acid screening test for the direct detection of WNV RNA in human plasma. The LoD for WNV L1 was determined at 6.06 IU/mL (95% CI: 5.1–7.7 IU/mL). For WNV L2, an international reference reagent was established by the World Health Organization (WHO) with no unit assigned.

The RealStar WNV RT-PCR Kit 2.0 is an in vitro diagnostic test, based on RT-PCR technology, for qualitative WNV-specific RNA detection. The manufacturer has not yet provided the LoD for WNV in IU/mL. The assay can be used with a variety of RNA extraction procedures and amplification platforms, as outlined in the package insert. For our analyses, the assay was validated using the QIAamp Viral RNA Mini Kit (Qiagen GmbH) and the LightCycler 480 Instrument II (Roche Diagnostics GmbH). In contrast to the cobas WNV assay, the RealStar WNV assay does not cross-react with other flaviviruses, as described by the manufacturer’s instructions for use.

A translation of crossing threshold and crossing point values into IU/mL was completed for both WNV assays, cobas and RealStar, respectively, via an external calibration curve using the first WHO international standard for WNV RNA (NIBSC code #18/206).

### Antibody testing

Anti-WNV IgM and IgG were detected using an enzyme-linked immunosorbent assay (ELISA) for IgM and IgG (Euroimmun Medizinische Labordiagnostika AG), respectively. The anti-WNV ELISA is coated with protein E and thus detects the standard antibody (Ab) WNV target. In addition, a non-structural protein 1 (NS1)-specific anti-WNV ELISA IgG (Euroimmun Medizinische Labordiagnostika AG) was performed to check for potential cross-reactivity with other flaviviruses.

According to manufacturer’s instructions for use, the results of all three ELISA tests were interpreted as reactive for WNV-specific Ab with a ratio of ≥ 1.1, borderline with a ratio between 0.8 and < 1.1 and not reactive with a ratio of < 0.8.

### Assessment of association with West Nile virus infection

The association between the blood donations and the WNV infection was determined using only the results obtained by the diagnostics performed at both BNITM and PEI.

Suspected WNV-positive cases were assessed in the first step as confirmed or not confirmed depending on whether the samples tested reactive for WNV or other flaviviruses via mNGS at BNITM, respectively. Therefore, considering the results obtained at PEI in the second step, a reactive ID-NAT test was required to classify the cases as likely, whereas a reactive serological test using at least one anti-WNV ELISA (IgM or IgG) was the criterion to evaluate the remaining cases as possible. The cases with borderline or not reactive results were classified as unlikely.

## Results

### Reporting of suspected West Nile virus-positive blood donations

A total of 26.4 million blood components were donated in Germany between 2020 and 2023.

**Figure 1:**
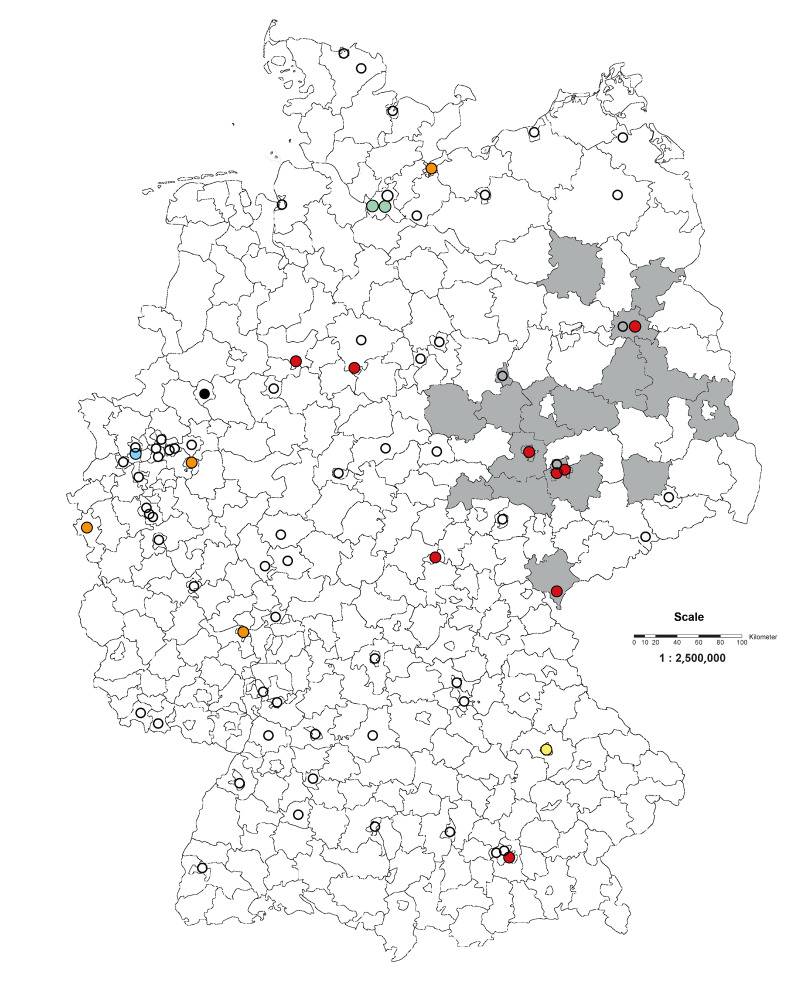
Geographical distribution within residence districts of blood establishments with or without suspected West Nile virus-positive blood donations, Germany, 2019–2023 (n = 77)

BE: blood establishment; WNV: West Nile virus.

During this period, 85 suspected WNV infections in blood donors were reported to the PEI by 19 of 77 BE located in 10 of 16 German states [24]. Except for one BE, all remaining 18 BE had at least one donor sample with sufficient volume for study analysis available and could therefore participate in the study. A total of 74 (87.1%) samples of the 85 suspected WNV-positive blood donations were sent to both BNITM and PEI within the observation period. The participating BE were located throughout Germany, both in and out of residence districts known to be WNV-endemic areas (Figure 1).

White circles: BE without blood samples collected for study analysis (n = 59); red circles: BE with at least one blood sample whose association with WNV infection was confirmed (n = 9); orange circles: BE with at least one blood sample whose association with WNV infection was likely (n = 4); yellow circles: BE with at least one blood sample whose association with WNV infection was possible (n = 1); green circles: BE with at least one blood sample whose association with WNV infection was unlikely (n = 2); blue circles: BE with at least one blood sample whose association with WNV infection was not confirmed (n = 1); black circles: BE with at least one blood sample whose association with WNV infection was not assessable (n = 1); grey area: residence district with at least one locally acquired human WNV infection according to the European Union case definition (Commission Implementing Decision (EU) 2018/945) reported under Decision No 2119/98/EC of the EU Parliament and of the Council.

Data source: GeoBasis-DE/BKG (2022); figure modified using: Photoshop (Adobe Inc.).

### Analysis of suspected West Nile virus-positive blood donors

Younger (18–39 years; n = 34, 45.9%) and older (≥ 40 years; n = 40, 54.1%) age groups were similarly represented (Table 1). With regard to sex, male (n = 47, 63.5%) donors were more frequently affected than female (n = 27, 36.5%) donors.

**Table 1:** Suspected West Nile virus-positive blood donors screened by blood establishments, Germany, 2020–2023 (n = 74)

Blood donor characteristics		Number of blood donors (n = 74)
Sex	Female	27
	Male	47
Age range in years	18–39	34
	40–59	22
	≥ 60	18
Stay in WNV-affected area^a^ in Germany	No	21
	Yes	34
	Unknown	19
Stay in WNV-affected area^a^ outside of Germany	No	26
	Yes	15
	Unknown	33
Time interval between suspected WNV-positive donation and previous WNV-negative donation in days	≤ 7	2
	8–21	7
	≥ 22	52
	Unknown	7
	Not applicable (first donation)	6
Number of donor samples pooled for MP-NAT	8–19	37
	20–48	17
	49–96	8
	Unknown	11
	Not applicable (only ID-NAT)	1

ID-NAT: individual donor nucleic acid amplification technique-based testing; MP-NAT: mini-pool nucleic acid amplification technique-based testing; WNV: West Nile virus.

^a^ Area with at least one locally acquired human WNV infection according to the European Union case definition (Commission Implementing Decision (EU) 2018/945) reported under Decision No 2119/98/EC of the EU Parliament and of the Council.

Berlin, Brandenburg, Sachsen, Sachsen-Anhalt and Thüringen were already known as WNV-affected areas having at least one officially confirmed WNV-positive human case during the season 2019 [14]. In addition, six more German states (Bayern, Hamburg, Niedersachsen, Nordrhein-Westfalen, Rheinland-Pfalz and Schleswig-Holstein) were reported as the residences of 21 (28.4%) suspected WNV-positive blood donors during the seasons under review (2020–23) (Table 1). A map is shown in Supplementary Figure S1.

Six (8.1%) and nine (12.2%) donors had temporarily resided in a foreign country with reported WNV outbreaks among humans or animals within 4 weeks and more than 4 weeks, respectively, before donation. However, 33 (44.6%) donors provided no information regarding the stay abroad, so a stay in a WNV-affected area outside Germany before donating blood cannot be excluded.

### Analysis of suspected West Nile virus-positive blood samples

Except for one donor sample that was not sequenced, mNGS at BNITM allowed the detection of WNV L2 RNA in 26 (35.6%) of 73 samples. Usutu virus (USUV) lineages Europe 2 and 3 were detected in three (4.1%) and six (8.2%) samples, respectively. Human pegivirus type 2 (HPgV-2) was identified in one (1.4%) sample and JEV vaccine strain RNA in three (4.1%) samples. A single (1.4%) case of coinfection with USUV Europe 3 and HPgV-2 was also identified (Table 2).

**Table 2:** Suspected West Nile virus-positive blood samples tested by health institutes, Germany, 2020–2023 (n = 74)

Screening tests	Interpretation of test results		Number of blood samples (n = 74)
NextSeq 2000 platform (Illumina Inc.)	Reactive	WNV L2 RNA	26
		USUV Europe 2 RNA	3
		USUV Europe 3 RNA	6
		HPgV-2 RNA	1
		Both USUV Europe 3 and HPgV-2 RNA	1
		JEV vaccine strain RNA	3
	Not reactive		33
	Not done		1
cobas WNV assay^a^ (Roche Diagnostics Inc.)	Reactive	Mean viral load in IU/mL: > 200	17
		Mean viral load in IU/mL: 20–200	13
		Mean viral load in IU/mL: < 20	15
	Not reactive		9
	Not done		20
RealStar WNV assay (altona Diagnostics GmbH)	Reactive	Mean viral load in IU/mL: > 20,000	3
		Mean viral load in IU/mL: 2,000–20,000	3
		Mean viral load in IU/mL: < 2,000	8
	Not reactive		35
	Not done		25
Anti-WNV ELISA IgM^a^ (Euroimmun Medizinische Labordiagnostika AG)	Reactive		10
	Borderline		1
	Not reactive		53
	Not done		10
Anti-WNV ELISA IgG^a^ (Euroimmun Medizinische Labordiagnostika AG)	Reactive		13
	Borderline		4
	Not reactive		47
	Not done		10
NS1-specific anti-WNV ELISA IgG (Euroimmun Medizinische Labordiagnostika AG)	Reactive		6
	Not reactive		58
	Not done		10

ELISA: enzyme-linked immunosorbent assay; HPgV-2: human pegivirus type 2; JEV: Japanese Encephalitis virus; L2: lineage 2; NS1: non-structural protein 1; USUV: Usutu virus; WNV: West Nile virus.

^a^ Including flavivirus cross-reactive specimens.

Health institutes: Bernhard Nocht Institute for Tropical Medicine (BNITM) and Paul-Ehrlich-Institut (PEI).

No sufficient amount of volume was available at PEI to perform for each donor sample both the cobas and the RealStar WNV assay; thus, the testing performed showed different results depending on the platform used. With the cobas WNV assay, 54 samples were ID-NAT-based tested: nine (16.7%) tested WNV not reactive, 17 (31.4%) had a viral load > 200 IU/mL, 13 (24.1%) had a viral load between 200 and 20 IU/mL and 15 (27.8%) had a viral load < 20 IU/mL. In contrast, 49 samples were tested with the RealStar WNV assay. The majority, 35 of 49 (71.5%) samples, were not reactive for WNV, three (6.1%) had a viral load > 20,000 IU/mL, three (6.1%) between 20,000 and 2,000 IU/mL and eight (16.3%) < 2,000 IU/mL.

No serology was performed in 10 donor samples because of insufficient specimen volume, regardless of anti-WNV ELISA used. Five (7.8%) of the remaining 64 samples were reactive in all three different ELISA performed, while four (6.2%) and six (9.4%) in at least two and one anti-WNV ELISA, respectively. Ab-borderline results were found with the anti-WNV ELISA IgM (n = 1, 1.6%) and anti-WNV ELISA IgG (n = 4, 6.2%). The remaining samples (n = 44, 68.8%) were not reactive in any of the anti-WNV ELISA performed.

### Evaluation of suspected West Nile virus-positive blood donations

**Figure 2:**
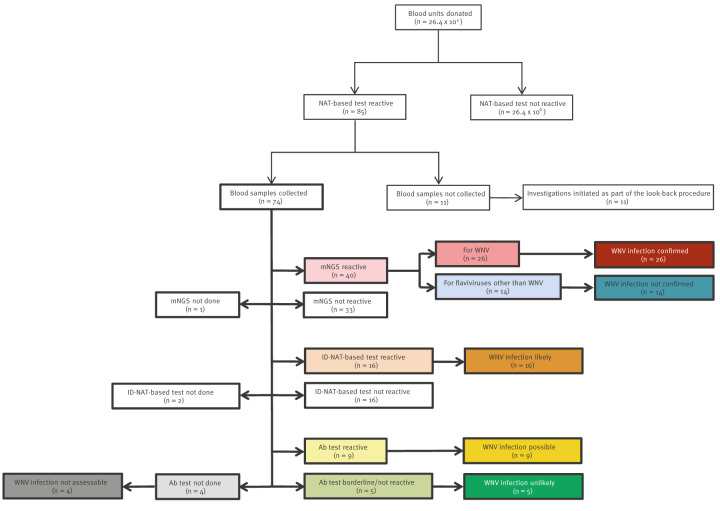
Assessment of association between blood donation and West Nile virus infection among blood donors, Germany, 2020–2023 (n = 74)

Of the 74 suspected WNV-positive blood donations examined, WNV-RNA was identified by mNGS in 26 (35.1%) cases, so the suspected WNV infection was classified as confirmed (Figure 2).

Ab: antibody; ID: individual donor; mNGS: metagenomic next-generation sequencing; NAT: nucleic acid amplification technique; WNV: West Nile virus.

With ca 6.6 million donations per year, this equates to an incidence of one suspected case per every ca 355,000 donations. However, in 14 (18.9%) cases, a WNV infection was not confirmed because mNGS only detected RNA from other flaviviruses. In 16 (21.6%) cases with WNV-reactive results from at least one NAT-based assay, the WNV infection was assessed as likely. The association with WNV infection in nine (12.2%) blood donations was classified as possible, specifically in five and four cases where WNV IgM and/or IgG were detected by all or at least one of the three ELISA performed, respectively. Five (6.8%) cases were defined as WNV unlikely as anti-WNV ELISA showed in the corresponding blood donations only Ab-borderline results or no reaction.

### Virus concentration and antibody detection in suspected West Nile virus-positive blood samples

The WNV concentration of repeat donors (n = 40, 66.7%) with a suspected late WNV-positive infection (previous WNV-negative donation ≥ 22 days) calculated with the cobas and the RealStar WNV NAT-assays showed median values of 90 IU/mL and 1,769 IU/mL, respectively. The 14 sequenced donor samples positive for flaviviruses other than WNV were not included in this calculation (Table 3).

**Table 3:** Viral load and antibody ratio detected during different incubation periods in suspected West Nile virus-positive blood samples, Germany, 2020–2023 (n = 60)

Screening tests	Interpretation of test results	Time interval between suspected WNV-positive donation and previous WNV-negative donation in days (number of blood samples)					
		≤ 7 (n = 2)	8–21 (n = 7)	≥ 22 (n = 40)	Unknown (n = 6)	Not applicable (first donation) (n = 5)	Total (n = 60)
NextSeq 2000 platform (Illumina Inc.)	Reactive	2	4	16	2	2	26
	Not reactive	0	3	23	4	3	33
	Not done	0	0	1	0	0	1
cobas WNV assay^a^ (Roche Diagnostics Inc.)	Reactive (median viral load in IU/mL)	2 (3,991)	3 (27)	22 (90)	2 (29.5)	5 (30)	34
	Not reactive	0	0	8	1	0	9
	Not done	0	4	10	3	0	17
RealStar WNV assay (altona Diagnostics GmbH)	Reactive (median viral load in IU/mL)	0 (not applicable)	2 (2,615)	9 (1,769)	1 (194)	2 (22,386)	14
	Not reactive	1	2	20	3	3	29
	Not done	1	3	11	2	0	17
Anti-WNV ELISA IgM^a^ (Euroimmun Medizinische Labordiagnostika AG)	Reactive	0	0	9	1	0	10
	Not reactive	2	7	26	2	5	42
	Not done	0	0	5	3	0	8
Anti-WNV ELISA IgG^a^ (Euroimmun Medizinische Labordiagnostika AG)	Reactive	0	1	10	1	0	12
	Borderline	0	0	3	0	1	4
	Not reactive	2	6	22	2	4	36
	Not done	0	0	5	3	0	8
NS1-specific anti-WNV ELISA IgG (Euroimmun Medizinische Labordiagnostika AG)	Reactive	0	0	5	1	0	6
	Not reactive	2	7	30	2	5	46
	Not done	0	0	5	3	0	8

ELISA: enzyme-linked immunosorbent assay; NS1: non-structural protein 1; WNV: West Nile virus.

^a^ Including flavivirus cross-reactive specimens.

Among repeat donors (n = 7, 11.7%) presumably infected by WNV within 2 weeks of a previous donation (previous WNV-negative donation between 8–21 days), the median viral load was 27 IU/mL in the cobas WNV assay and 2,614.5 IU/mL in the RealStar WNV assay. The measurement of WNV concentration in repeat donors (n = 2, 3.3%) with a suspected early WNV-positive infection (previous WNV-negative donation ≤ 7 days) was only possible with the cobas WNV assay resulting in a median value of 3,990 IU/mL.

All reactive serological results were obtained from testing donor samples with suspected late WNV-positive infections, irrespective of the anti-WNV ELISA performed. The one exception was represented by a donor sample collected between the second and third week after the previous WNV-negative donation that showed a reactive result with the anti-WNV ELISA IgG.

## Discussion

This article aimed to investigate the effectiveness of German regulatory RMM to prevent WNV transfusion-transmitted infections. Over a period of 4 years (2020–23), 74 suspected WNV-positive blood donations were reported in Germany. All affected donors were asymptomatic at time of donation. However, suspected cases of WNV transfusion-transmitted infection have never been reported. Even if it can be assumed that a WNV transfusion-transmitted infection is not clinically or experimentally detectable in every case, the risk of WNV transfusion-transmitted infection can currently be considered low.

According to German regulations, the NAT-based method employed by BE for WNV screening should have a minimum LoD of 120 IU/mL calculated for a single donation. This LoD could detect most suspected WNV-positive donor samples at the screening stage, according to previous experience gained in the United States (US), Canada and Italy [25-29]. Detection of WNV-reactive samples was possible at a virus concentration below 20 IU/mL in an MP of 48 blood donors using the cobas WNV assay. After sample enrichment, the same assay was also able to detect a virus concentration below 20 IU/mL in an MP of 96 donors. The sensitivity of the RealStar WNV assay was lower mainly due to the reduced sample volume applied. An 1,800 IU/mL virus concentration was detected in an MP of 48 donors. Thus, it is important to note that the reliable detection of WNV- as well as USUV-specific RNA often requires sample enrichment because of the typically low viral load present in blood donations. This highlights a potential limitation in assessing the true prevalence of both pathogens in our blood supply and suggests that current detection methods may need to be refined or replaced by more sensitive screening methods to ensure comprehensive monitoring [30].

Using the mNGS, BNITM confirmed a WNV L2 infection in 26 of 73 suspected WNV-positive donations sequenced corresponding to a confirmation rate of 35.6%. WNV L1 RNA was not identified in any of the samples analysed, whereas USUV Europe 2 and 3 RNA was detected in a notable percentage of cases (13.7%). While our findings in Germany primarily identified WNV RNA in blood donations, in neighbouring countries such as Austria, USUV RNA was detected more frequently in blood donations than WNV RNA, stressing the relevance of both flaviviruses as emerging pathogens of concern for public health in Europe and hence underscoring the necessity for continuous epidemiological surveillance [30,31]. Confirmed WNV infections occurred predominantly in donors who stayed in national or international WNV-endemic areas. This distinct regional variation of distribution of confirmed WNV-positive cases likely reflects differences in local vector populations and environmental conditions conducive to WNV transmission. Like WNV, also USUV shows a distinct epidemiological pattern across the European continent. While WNV has been associated with large outbreaks, particularly in southern and eastern Europe, USUV has been increasingly detected in central European regions, including Germany [30,31]. Notably, multiple lineages of both viruses circulate within Europe, with WNV L1 and L2 and USUV Europe 2 and 3 being the most prevalent [15,30]. The presence of these different lineages underscores the need for targeted surveillance and intervention strategies to mitigate the risk of these viruses entering the blood supply. In five donor samples with an initial WNV-reactive NAT-based result obtained by BE, mNGS detected the presence of an infection caused by HPgV-2 or a JEV vaccine strain. Although NAT-based tests demonstrate high sensitivity for WNV, they also show broad cross-reactivity to other flaviviruses, including USUV, JEV, Kunjin virus, Murray Valley encephalitis virus and Saint Louis encephalitis virus [32]. The singular HPgV infection in one NAT-reactive donor sample raises questions about the potential for other flaviviruses, besides those mentioned in the literature, to cross-react in NAT-based tests. This finding requires careful consideration, as there is currently no evidence of such cross-reactivity, and further investigations are necessary to clarify this. In addition, it should be considered that the sample storage at 4 °C during the time interval between initial NAT and mNGS potentially caused RNA degradation. This may have contributed to a reduction of mNGS sensitivity and therefore explain the discrepancy between the reactive MP-NAT result obtained by BE and the not reactive mNGS result obtained by BNITM. A low virus concentration was indeed found at PEI in most of the 15 samples with a reactive ID-NAT-based result and a not reactive mNGS result.

At PEI, 54 of the 74 suspected WNV-positive blood donations could be tested by ID-NAT with the cobas WNV assay. In 45 samples, the WNV-reactive NAT-based result obtained by BE during donor screening was confirmed. Among the remaining 20 suspected WNV-positive donations, three additional samples showed a reactive ID-NAT-based result with the RealStar WNV assay. These data support the assumption that a sufficiently reliable donor screening is guaranteed with a sensitive NAT-based test. Because of the overall low number of WNV-positive blood donors in Germany, a larger percentage of non-specific or false-reactive test results at BE could be tolerated. In these cases, the blood donors would be temporarily deferred from donation until a NAT-based test result not reactive for WNV during screening would be available. In the ID-NAT performed with the RealStar WNV assay at PEI on 49 samples, 14 were reactive, of which 12 also showed a positive result with mNGS. This corresponds to a 28.6% confirmation rate among the suspected WNV-positive blood donations collected. Despite its low sensitivity in our testing regimen, a WNV NAT-based test with high specificity seems appropriate as a confirmatory test. In case of a reactive result, a suspected WNV infection can be considered likely. However, we believe there is potential for improvement by implementing a WNV-specific NAT-based test with greater sensitivity for confirmation.

The results of the anti-WNV ELISA reflected the possible test sensitivity about the timing of suspected WNV infection. Among the donor samples screened WNV-reactive by MP-NAT with confirmed WNV infection by mNGS, only one sample, which was collected 4 weeks after the previous WNV-negative donation, tested reactive for anti-WNV IgG. All other mNGS confirmable specimens were not reactive for anti-WNV Ab, including samples from more recent WNV infections (within 3 weeks after the previous WNV-negative donation), or at most showed an Ab-borderline test result. This observation aligns with the expected timeline for early WNV detection in serum/plasma using MP-NAT, typically occurring within 2–8 days of infection, and subsequent serological detection of IgM and IgG, usually ca 3.9–7.7 days later [33]. Interestingly, this expected timing for seroconversion was observed in two of three blood donations with available follow-up samples. In a first case, the test result changed from serologically non-reactive (6 days after the previous WNV-negative donation) to reactive (13 days after the previous WNV-negative donation) within 7 days. In a second case (first donation), an Ab-borderline test result was followed by an Ab-reactive test result (interval range unknown). Seven of nine donations screened were WNV-reactive in a MP-NAT test, were not confirmed to be infected with WNV by mNGS and ID-NAT, but were reactive to anti-WNV IgM and/or IgG. Their documented time interval between the suspected WNV-positive donation and the previous WNV-negative donation exceeded 28 days. It can be assumed that these donors showed a residual low-level viraemia, which was still detectable during screening only with a sensitive MP-NAT assay, and an already developed immune response at time of donation. No or only small amounts of viral RNA were detectable in a long-standing WNV infection with successful seroconversion as described in the literature [34]. Based on the performance of these serological test data [35], the benefit of WNV donor screening by serology appears therefore limited. Isolated reactive serological test results thereby confirm a longer existing WNV infection, but the risk of transfusion-related viral transmission can be classified as very low in these cases. Routine serological WNV donor testing does not therefore guarantee a higher safety standard.

As mentioned previously, no WNV transfusion-transmitted infections were reported in Germany from 2020 to 2023. In 2003, the US Centers for Disease Control and Prevention described the results of WNV donor screening and the incidence of WNV transfusion-associated transmission [36]. From June through December 2003, approximately six million blood units were screened for WNV and at least 818 viraemic donations were removed from the blood supply, which corresponded to an incidence of one viraemic donation per every ca 7,335 blood units. Six of 23 cases reported were classified as confirmed or probable WNV transfusion-associated transmissions, 11 as non-cases and three as inconclusive. Current data on WNV transfusion-associated transmission frequency in the US or Europe are not available and only sporadic WNV transfusion-transmitted infections have been documented worldwide. Lastly, the WNV concentration of the 74 collected samples measured with the cobas WNV assay was between 47,545 IU/mL and < 20 IU/mL. Little information in the literature on the WNV infectivity of blood samples with a low virus concentration should be noted [37]. Because of the lack of reliable data on the infectivity of blood components, the WNV load necessary to cause transmission or infection in a blood recipient is currently unknown. 

While the laboratory tests were conducted by the authors, the analysis of the donor data is based on information provided by the BE, which the authors cannot verify. Furthermore, the BE participated in the study on a voluntary basis, so it must be assumed that the data collected during the analysed period do not represent the complete dataset at the national level. Therefore, it must be assumed that the reported suspected WNV-positive blood donations are underestimated. However, this limitation does not have a substantial impact on the statement regarding the benefit of the RMM.

## Conclusion

So far in Germany, no WNV transfusion-transmitted infection has been reported, and the established donor screening has found only a few (1:355,000) suspected WNV-positive blood donations. The prescribed 120 IU/mL LoD for WNV NAT-based donor screening seems to be sufficient to detect the viral genome in the early and late stages of infection, even at low virus concentrations in MP with 48 or more blood donations. In contrast, serological tests do not represent a valid alternative diagnostic tool for WNV screening as they have shown positive results almost exclusively 28 days after infection. The RMM in place can therefore currently be considered sufficient. However, constant monitoring is recommended as the WNV endemic is expected to spread in Germany.

## Supplementary Data

498000 Supplement
