# Supplementary material for: Assessment of the effectiveness of West Nile virus screening by analysing suspected positive donations among blood donors, Germany, 2020 to 2023
Source: Euro Surveill. 2025 Feb 27;30(8):2400373. doi: 10.2807/1560-7917.ES.2025.30.8.2400373 (PMC11869365; doi:10.2807/1560-7917.ES.2025.30.8.2400373)

**SUPPLEMENTARY FIGURE S1.** Geographical distribution within federal states of residence districts with at least one locally acquired human West Nile virus infection according to the European Union case definition (Commission Implementing Decision (EU) 2018/945) reported under Decision No 2119/98/EC, Germany, 2019–2024 (n = 29)

This supplementary material is hosted by *Eurosurveillance* as supporting information alongside the article Assessment of the effectiveness of West Nile virus screening by analysing suspected positive donations among blood donors, Germany, 2020 to 2023, on behalf of the authors, who remain responsible for the accuracy and appropriateness of the content. The same standards for ethics, copyright, attributions and permissions as for the article apply. Supplements are not edited by *Eurosurveillance* and the journal is not responsible for the maintenance of any links or email addresses provided therein.

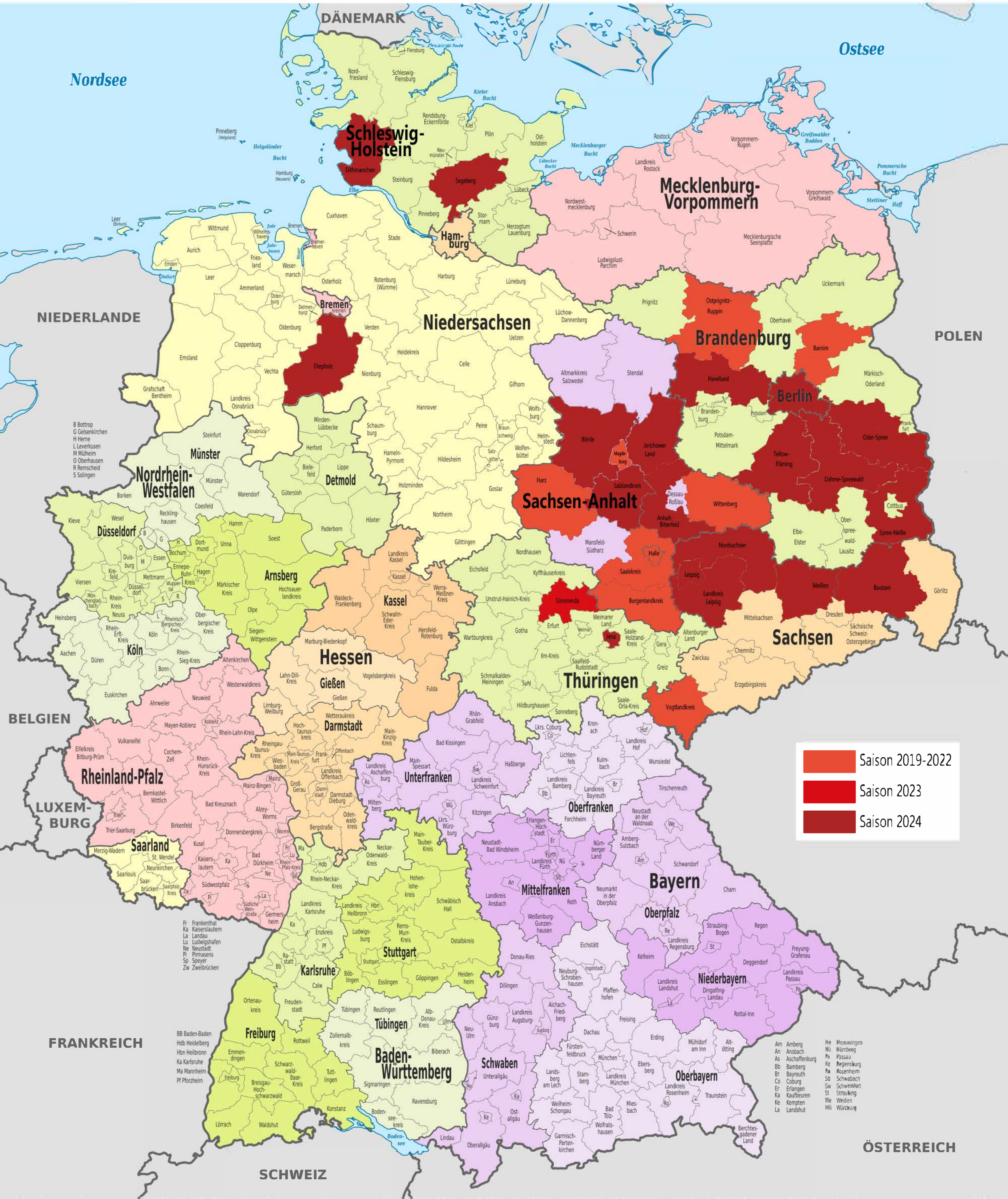

Supplement: Supplement [file 24-00373_FUNK_Supplement.pdf]
